# Spectral-Based Blind Image Restoration Method for Thin TOMBO Imagers

**DOI:** 10.3390/s8096108

**Published:** 2008-09-26

**Authors:** Amar A. El-Sallam, Farid Boussaid

**Affiliations:** School of Electrical, Electronic and Computer Engineering, The University of Western Australia, 35 Stirling Highway, Crawley, WA 6009, Australia

**Keywords:** Image Restoration, TOMBO, CMOS Imager, Back-Projection, Cross-correlation, Spectra

## Abstract

With the recent advances in microelectronic fabrication technology, it has now become now possible to fabricate thin imagers, less than half a millimeter thick. Dubbed TOMBO (an acronym for Thin Observation Module by Bound Optics), a thin camera-on-a-chip integrates micro-optics and photo-sensing elements, together with advanced processing circuitry, all on a single silicon chip. Modeled after the compound-eye found in insects and many other arthropods, the TOMBO imager simultaneously captures a mosaic of low resolution images. In this paper, we describe and analyze a novel spectral-based blind algorithm that enables the restoration of a high resolution image from the captured low resolution images. The proposed blind restoration method does not require prior information about the imaging system nor the original scene. Furthermore, it alleviates the need for conventional de-shading and rearrangement processing techniques. Experimental results demonstrate that the proposed method can restore images for Signal-to-Noise Energy Ratio (SNER) lower than 3 dB.

## 1. Introduction

**Figure 1.**
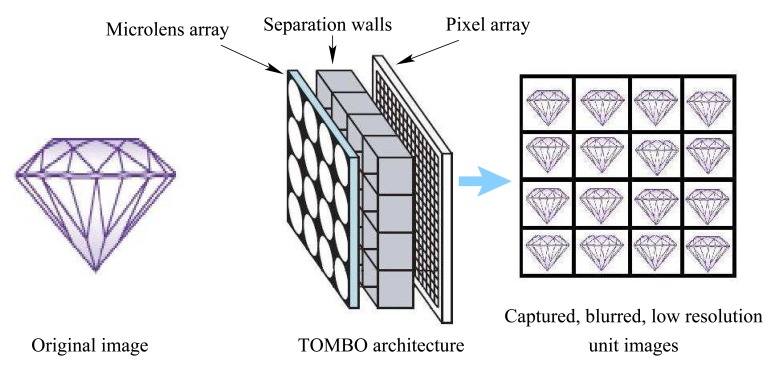
The TOMBO architecture

Rapid advances in semiconductor CMOS fabrication technology have enabled the realization of the concept of a camera-on-a-chip, i.e., a CMOS imager [1]. The integration of an increased number of camera functions onto a single silicon chip offers significant advantages in terms of system miniaturization and manufacturing cost [1]. As a result, CMOS imagers can now be found in a wide range of consumer electronic products from mobile phones, PC mice and webcams to fax machines, to name a few. Other examples of applications of CMOS imagers include blind spot detection and rear vision in cars, automated industrial product quality inspection, photography, digital radiography, fluoroscopy, microscopy and even implantable microimager retinal stimulation [2]. CMOS camera manufacturers have been continuously improving the performance of their products in terms of resolution, power consumption, and read-out speed [1]. The continuous aggressive scaling of the minimum feature size in CMOS technology offers the possibility to further miniaturize CMOS imagers, while still integrating increased built-in functionalities and advanced on-chip processing for improved imaging performance [1]. However, a further miniaturization of the optics is not possible since this would cause light to pass through a very small opening and to diffract or spread due to the interference of light waves. Diffraction would cause so much spreading that the image would be very blurry and essentially useless. To achieve a thin camera-on-a chip, Tanida *et al* have recently proposed an imaging system called TOMBO [3] (an acronym for Thin Observation Module by Bound Optics) emulating the visual system of insects and other arthropods [4, 5]. In contrast to traditional camera systems, TOMBO is not based on a single lens system [3]. Instead, a TOMBO imager comprises a collection of imaging units, each of which consists of a microlens unit associated to a subset of the pixel array (Fig. 1). Adjacent imaging units are separated by an opaque wall to prevent crosstalk. Each individual imaging unit is thus optically isolated and images part of the scene. As a result, the TOMBO sensor captures multiple low resolution (LR) images at the same time and the output is a compound image formed by the mosaic of low resolution unit images. Advanced Digital signal processing can then be used to reconstruct a high resolution image from the available set of low resolution images [3, 10]. Tanida *et al* have demonstrated that the restoration of a high resolution image is possible since each imaging unit provides a different view of the scene [7]-[12]. In [3], Tanida *et al* proposed an image restoration algorithm which uses the back-projection (BP) method. This algorithm requires complete knowledge of the imaging system point spread function (PSF). To reconstruct the original image, the inverse (pseudo-inverse) of the known PSF is multiplied by the captured low resolution images. This approach has a number of limitations: (i) the PSF differs from one camera to another and cannot be easily identified from the system parameters [10] (ii) poor performance when used in an experimental TOMBO system, due to the variation in the properties of the individual imaging units [10] (iii) the matrix representing the PSF can be singular, i.e. non-invertible.

**Figure 2.**
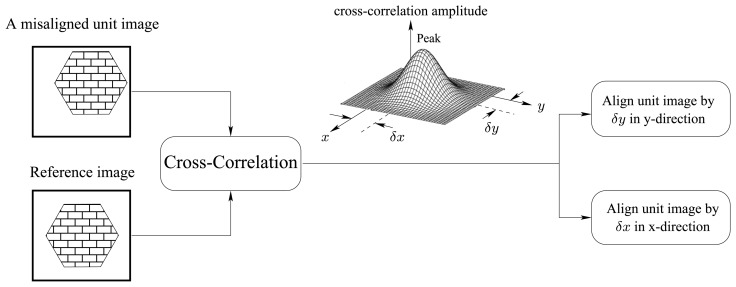
Cross correlation-based pixel rearrange method

To overcome the above limitations, Tanida *et al* proposed a new image reconstruction approach called, pixel rearrange method [10], which could be integrated to enable the realization of a compact, low cost thin imaging system. In their approach, a cross-correlation based technique is used to arrange and align unit image pixels. To correct for the misalignment, a unit reference image is used. The relative shift values (*δx* and *δy* in Fig. 2) of each unit image with respect to the reference image are determined by identifying the peak location of the cross-correlation function between the unit image and the reference one. Interpolation techniques were used to identify the cross-correlation peak [10]. The cross-correlation based pixel rearrange method is illustrated in Fig. 2.

In the rearrangement process, it is assumed that the cross-correlation function is ideally symmetric around a single peak. In other words, there is a single shift between the considered unit image and the reference image, i.e., the spatial PSF function has only one parameter. In reality, however, there would be more than one parameter in a PSF (i.e., several cross-correlation peaks). This will limit the performance of the rearrange method when aligning unit images. Furthermore, the presence of several cross-correlation peaks introduces additional blur in the restored image. Inverse filtering is subsequently required. This operation is not only computationally costly but also unstable if at least a single non-minimum phase component is present. It also requires for the PSF to be known. Besides the spatial PSF, additive noise can also introduce false cross-correlation peaks, which further degrades significantly the performance of rearrange method [10].

In the same paper, Tanida *et al* proposed a method to minimize the problems associated with: (i) TOMBO's intrinsic PSFs (ii) imager internal noise, and (iii) shading introduced by the separating walls (Fig. 1). To overcome these problems, Tanida *et al* introduced a de-shading pre-processing step, which uses a black picture and a white one for calibration. We can analyze the de-shading process by noting that,

(1) $$B(x,y)=h_{\text{int}}(x,y)B_{i}(x,y)+V_{B}(x,y)$$

(2) $$W(x,y)=h_{\text{int}}(x,y)W_{i}(x,y)+V_{W}(X,Y)$$

where, *x* and *y* define the pixel location, *h_int_*(*x*, *y*) represents the intrinsic PSF of the TOMBO imager, *B_i_*(*x*, *y*) and *W_i_*(*x*, *y*) are the black and white pictures to be captured, *B*(*x*, *y*) and *W*(*x*, *y*) are the captured black and white images, and *V_B_*(*x*, *y*) and *V_W_*(*x*, *y*) are the additive internal noise for the black and white images respectively.

By subtracting Eqn. (1) from Eqn. (2) and after some manipulation we have,

(3) $$h_{\text{int}}(x,y)=\frac{\left(W(x,y)-B(x,y))-(V_{W}(x,y)-V_{B}(x,y)\right)}{W_{i}(x,y)-B_{i}(x,y)}$$

When normalized, a black pixel correspond to a ‘0’ value while a white pixel has a value of ‘1’, i.e., *B_i_*(*x*, *y*) = 0 and *W_i_*(*x*, *y*) = 1, thus

(4) $$\begin{array}{ccc} h_{\text{int}}(x,y)=(W(x,y)-B(x,y))-(V_{W}(x,y)-V_{B}(x,y)) & \text{and} & B(x,y)=V_{B}(x,y) \end{array}$$

Tanida *et al* assume *V_W_*(*x, y*) and *V_B_*(*x, y*) to be equal. As a result, the intrinsic PSF will take the form,

(5) $$h_{\text{int}}(x,y)=W(x,y)-B(x,y)$$

**Figure 3.**
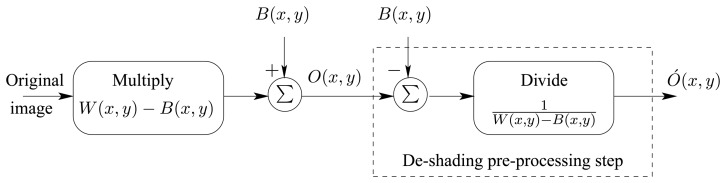
A model for the shading and de-shading pre-processing step

The model representing the intrinsic characteristics and the de-shading pre-processing step can be seen in Fig. 3.

When an image *O*(*x*, *y*) is captured, the de-shading pre-processing step (Fig. 3) generates the image:

(6) $$\overset{´}{O}(x,y)=C\frac{O(x,y)-B(x,y)}{W(x,y)-B(x,y)}$$

where *C* > 0 is an amplification factor.

From the above equations, it is clear that dividing by the intrinsic PSF can introduce nonlinearity problems and unrealistic large pixel values in the de-shaded image. Furthermore, it is assumed that the internal noises *V_W_*(*x*, *y*) and *VB*(*x*, *y*) are equal while in reality they are not and can differ from one image to another and from one imager to another.

In this paper, a spectral-based blind image restoration algorithm is proposed for TOMBO thin imagers [13]. The mathematical analysis for the algorithm is conducted in the frequency domain. Unlike conventional TOMBO image restoration methods [3] and [7]-[12], our method:

- does not require prior information about the imaging system (e.g. intrinsic PSF and internal noise)
- does not require prior information about the original scene and the PSFs (mapping the original scene into the pixel array)
- is robust in the presence of additive noise whether external, internal, correlated or uncorrelated and at low signal to noise ratios (SNR)
- does not require a de-shading pre-processing step
- does not require pixel rearrangement since this is automatically done in the frequency domain through the restoration process
- is able to restore original images that are blurred without the use of inverse filtering
- requires less than 30 iterations at SNRs ≤ 7 dB to restore the original image

The paper is organized as follows. Section 2 gives a model of the TOMBO system. Section 3 describes the proposed spectral-based image restoration approach. Section 4 presents the blind image restoration algorithm. The experimental results are discussed in Section 5. Finally, a conclusion is given in Section 6.

## 2. System Model

If the original image can be regarded as either a deterministic or stochastic signal and if the cause of image degradation can be modeled, then the original image can be reconstructed. However, in real situations, it is difficult to accurately model image degradation since it has many origins: atmospheric turbulences, an out-of-focus camera, relative motion between the camera and the scene, electronic imaging components, the number of pixels used to capture the image, to name a few. In other words, an image can be degraded or blurred by a linear or nonlinear process, distorted by an additive or multiplicative noise process [16].

Consider the TOMBO system shown in Fig. 1 with a (*μ* × *μ*) imaging units. Each captured unit image can be modeled by,

(7) $$\begin{array}{cc} g_{i,j}(x,y)=[h_{i,j}(x,y)**f(x,y)+v_{i,j}(x,y)]↓D, & i,j=1,2,\ldots ,\mu \end{array}$$

Where,

- *g_i,j_*(*x,y*) represents the captured (*i,j*) blurred, low resolution (LR) and noisy unit image with dimension (*M* × *N*) pixels
- *h_i,j_*(*x*, *y*) is a two-dimensional (2-d) PSF with dimension (ℓ × ℓ), representing the channel blur for the (*i*, *j*) image unit
- “* *” represents the 2-d convolution operator
- *f*(*x*, *y*) is the original image with resolution (*M* × 
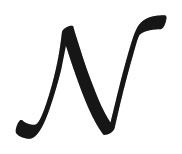
)
- *v_i,j_*(*x*, *y*) is the additive 2-d zero mean white Gaussian noise introduced when capturing the (*i*, *j*) unit image
- ↓ *D* is the down-sampling factor modeling the LR operation done to the captured images

**Figure 4.**
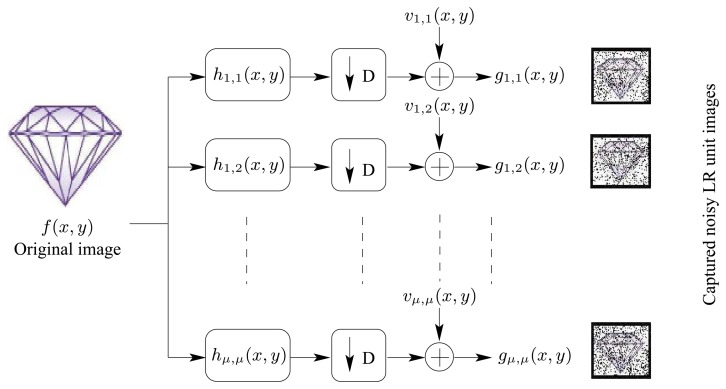
A mathematical model for the TOMBO system

The overall system model is illustrated in Fig. 4

## 3. Spectral-Based Image Restoration Approach

Our objective is to restore or estimate the original image *f*(*x*, *y*), given only the blurred, LR and noisy images *g_i,j_*(*x*, *y*), *i*, *j* = 1,2,…,*μ* captured by the TOMBO. This objective should to be achieved with minimal information about the original image, PSFs and noise contribution. To tackle the problem, we propose a spectral-based image restoration technique. We consider the 2-d z-transform of the model given by Eqn. (7), i.e.,

(8) $$G_{i,j}(z_{1},z_{2})=\frac{1}{D^{2}}\sum_{k=0}^{D-1}\sum_{l=0}^{D-1}H_{i,j}\left(z_{1}^{\frac{1}{D}}e^{\frac{-j2\pi k}{D}},z_{2}^{\frac{1}{D}}e^{\frac{-j2\pi l}{D}}\right)F\left(z_{1}^{\frac{1}{D}}e^{\frac{-j2\pi k}{D}},z_{2}^{\frac{1}{D}}e^{\frac{-j2\pi l}{D}}\right)+V_{i,j}\left(z_{1}^{\frac{1}{D}}e^{\frac{-j2\pi k}{D}},z_{2}^{\frac{1}{D}}e^{\frac{-j2\pi l}{D}}\right),$$

where, *z*_1_ = *e*^−^*^j^*^2^*^πf^*^1^ and *z*_2_ = *e*^−^*^j^*^2^*^πf^*^2^.

**Figure 5.**
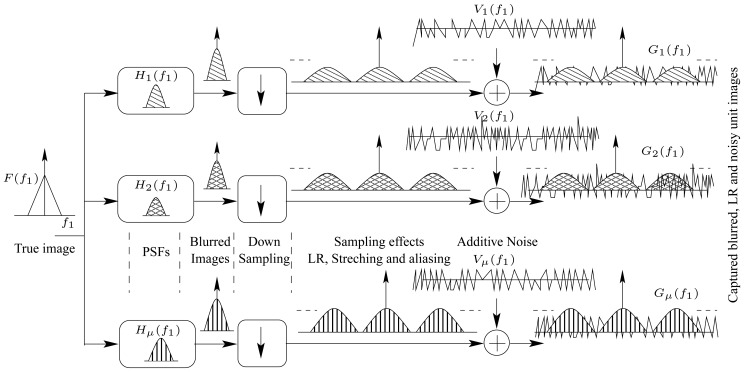
A spectral representation for the captured images in the TOMBO system.

Fig. 5 illustrates the spectra of all involved signals. For the sake of clarification, the spectra are shown for one-dimension signals. The figure also shows the impact of both the down-sampling operator, “↓” and the noise in degrading the original image.

Eqn. (8) can be partitioned into,

(9) $$\begin{array}{lll} G_{i,j}(z_{1},z_{2}) & = & \underset{\left(\text{a}\right)}{\underset{︸}{\frac{1}{D^{2}}\left[H_{i,j}\left(z_{1}^{\frac{1}{D}},z_{2}^{\frac{1}{D}}\right)F\left(z_{1}^{\frac{1}{D}},z_{2}^{\frac{1}{D}}\right)+V_{i,j}\left(z_{1}^{\frac{1}{D}},z_{2}^{\frac{1}{D}}\right)\right]}}\\ & + & \underset{\left(\text{b}\right)}{\underset{︸}{\frac{1}{D^{2}}\sum_{k=1}^{D-1}\sum_{l=1}^{D-1}H_{i,j}\left(z_{1}^{\frac{1}{D}}e^{\frac{-j2\pi k}{D}},z_{2}^{\frac{1}{D}}e^{\frac{-j2\pi l}{D}}\right)F\left(z_{1}^{\frac{1}{D}}e^{\frac{-j2\pi k}{D}},z_{2}^{\frac{1}{D}}e^{\frac{-j2\pi l}{D}}\right)}}\\ & + & \underset{\left(\text{c}\right)}{\underset{︸}{\frac{1}{D^{2}}\sum_{k=1}^{D-1}\sum_{l=1}^{D-1}V_{i,j}\left(z_{1}^{\frac{1}{D}}e^{\frac{-j2\pi k}{D}},z_{2}^{\frac{1}{D}}e^{\frac{-j2\pi l}{D}}\right)}} \end{array}$$

and can be rewritten as,

(10) $$\begin{array}{lll} G_{i,j}(z_{1},z_{2}) & = & \frac{1}{D^{2}}\left[H_{i,j}\left(z_{1}^{\frac{1}{D}},z_{2}^{\frac{1}{D}}\right)F\left(z_{1}^{\frac{1}{D}},z_{2}^{\frac{1}{D}}\right)+V_{i,j}\left(z_{1}^{\frac{1}{D}},z_{2}^{\frac{1}{D}}\right)\right]\\ & + & T_{i,j}^{b}\left(z_{1}^{\frac{1}{D}},z_{2}^{\frac{1}{D}}\right)\\ & + & T_{i,j}^{c}\left(z_{1}^{\frac{1}{D}},z_{2}^{\frac{1}{D}}\right) \end{array}$$

where,

- (a) represents the image of interest and the noise term, in-band useful terms
- (b) symbolized by $T_{i,j}^{b}\left(z_{1}^{\frac{1}{D}},z_{2}^{\frac{1}{D}}\right)$ represents the aliasing out-off band image terms
- (c) symbolized by $T_{i,j}^{c}\left(z_{1}^{\frac{1}{D}},z_{2}^{\frac{1}{D}}\right)$ represents the aliasing out-off band noise term

By multiplying both sides of Eqn. (10) with the complex conjugate of $H_{i,j}\left(z_{1}^{\frac{1}{D}},z_{2}^{\frac{1}{D}}\right)$, i.e., $H_{i,j}^{*}\left(z_{1}^{\frac{1}{D}},z_{2}^{\frac{1}{D}}\right)$ and applying the ensemble average, E {} and after some manipulation we have,

(11) $$\begin{array}{lll} \text{E}\left\{G_{i,j}H_{i,j}^{*}(z_{1},z_{2})\right\} & = & \frac{1}{D^{2}}\left[\text{E}\left\{F\left(z_{1}^{\frac{1}{D}},z_{2}^{\frac{1}{D}}\right)H_{i,j}H_{i,j}^{*}\left(z_{1}^{\frac{1}{D}},z_{2}^{\frac{1}{D}}\right)\right\}+\text{E}\left\{V_{i,j}H_{i,j}^{*}\left(z_{1}^{\frac{1}{D}},z_{2}^{\frac{1}{D}}\right)\right\}\right]\\ & + & \text{E}\left\{T_{i,j}^{b}H_{i,j}^{*}\left(z_{1}^{\frac{1}{D}},z_{2}^{\frac{1}{D}}\right)\right\}\\ & + & \text{E}\left\{T_{i,j}^{c}H_{i,j}^{*}\left(z_{1}^{\frac{1}{D}},z_{2}^{\frac{1}{D}}\right)\right\} \end{array}$$

which can rewritten as,

(12) $$\begin{array}{lll} C_{G_{i,j}H_{i,j}^{*}}(z_{1},z_{2}) & = & \frac{1}{D^{2}}\left[F\left(z_{1}^{\frac{1}{D}},z_{2}^{\frac{1}{D}}\right)C_{H_{i,j}H_{i,j}^{*}}\left(z_{1}^{\frac{1}{D}},z_{2}^{\frac{1}{D}}\right)+C_{V_{i,j}H_{i,j}^{*}}\left(z_{1}^{\frac{1}{D}},z_{2}^{\frac{1}{D}}\right)\right]\\ & + & C_{T_{i,j}^{b}H_{i,j}^{*}}\left(z_{1}^{\frac{1}{D}},z_{2}^{\frac{1}{D}}\right)\\ & + & C_{T_{i,j}^{c}H_{i,j}^{*}}\left(z_{1}^{\frac{1}{D}},z_{2}^{\frac{1}{D}}\right) \end{array}$$

Where the cross-spectra C_XY__*_(*z*_1_, *z*_2_) = E{*X*(*z*_1_, *z*_2_)Y*(*z*_1_, *z*_2_)} [13]

Since $T_{i,j}^{b}\left(z_{1}^{\frac{1}{D}},z_{2}^{\frac{1}{D}}\right)$ and $T_{i,j}^{c}\left(z_{1}^{\frac{1}{D}},z_{2}^{\frac{1}{D}}\right)$ are not located in the same frequency band where $H_{i,j}^{*}\left(z_{1}^{\frac{1}{D}},z_{2}^{\frac{1}{D}}\right)$ is located, thus the second and the last cross-spectral terms $C_{T_{i,j}^{b}H_{i,j}^{*}}\left(z_{1}^{\frac{1}{D}},z_{2}^{\frac{1}{D}}\right)$ and $C_{T_{i,j}^{c}H_{i,j}^{*}}\left(z_{1}^{\frac{1}{D}},z_{2}^{\frac{1}{D}}\right)$ in Eqn. (12) will disappear, leading to,

(13) $$C_{G_{i,j}H_{i,j}^{*}}(z_{1},z_{2})=\frac{1}{D^{2}}\left[F\left(z_{1}^{\frac{1}{D}},z_{2}^{\frac{1}{D}}\right)C_{H_{i,j}H_{i,j}^{*}}\left(z_{1}^{\frac{1}{D}},z_{2}^{\frac{1}{D}}\right)+C_{V_{i,j}H_{i,j}^{*}}\left(z_{1}^{\frac{1}{D}},z_{2}^{\frac{1}{D}}\right)\right]$$

Similarly and by following the same previous procedure,

(14) $$C_{G_{i,j}F^{*}}(z_{1},z_{2})=\frac{1}{D^{2}}\left[H_{i,j}\left(z_{1}^{\frac{1}{D}},z_{2}^{\frac{1}{D}}\right)C_{FF^{*}}\left(z_{1}^{\frac{1}{D}},z_{2}^{\frac{1}{D}}\right)+C_{V_{i,j}F^{*}}\left(z_{1}^{\frac{1}{D}},z_{2}^{\frac{1}{D}}\right)\right]$$

where $C_{V_{i,j}H_{i,j}^{*}}\left(z_{1}^{\frac{1}{D}},z_{2}^{\frac{1}{D}}\right)$ and $C_{V_{i,j}F^{*}}\left(z_{1}^{\frac{1}{D}},z_{2}^{\frac{1}{D}}\right)$ are residual errors representing the in-band cross spec-tral terms between original image, PSFs and the independent additive noise in Eqn. (11) respectively. This error can be assumed to be a zero mean, 2-d independent and identically distributed signal (i.i.d) under some regularity conditions. In addition, these error terms represent the cross-spectra between signals and noise, i.e. smaller in amplitude when compared with the spectra of the signals themselves [14].

Eqns. (13) and (14) suggest that it is possible to estimate the original image and the PSFs with minimal prior information/constraints on the PSFs/original image. For instance, if the PSF associated with each (*i*, *j*) unit image, $H_{i,j}\left(z_{1}^{\frac{1}{D}},z_{2}^{\frac{1}{D}}\right)$ is known or can be estimated, then the original image can be restored using,

(15) $$F\left(z_{1}^{\frac{1}{D}},z_{2}^{\frac{1}{D}}\right)=\frac{C_{G_{i,j}H_{i,j}^{*}}\left(z_{1},z_{2}\right)-\frac{1}{D^{2}}C_{V_{i,j}H_{i,j}^{*}}\left(z_{1}^{\frac{1}{D}},z_{2}^{\frac{1}{D}}\right)}{\frac{1}{D^{2}}C_{H_{i,j}H_{i,j}^{*}}\left(z_{1}^{\frac{1}{D}},z_{2}^{\frac{1}{D}}\right)}$$

However, there are three main problems when using image and PSF estimation methods in real situations. The first is the unknown size of the PSF. The second is the PSF itself, and the third and mostcritical one, is the residual error term, which can significantly affect the restoration process [15]-[22].

Since TOMBO imaging systems provide multiple observations of the scene, it is possible to significantly reduce the impact of the error terms using averaged cross-spectral techniques [13]. To demonstrate this, consider a TOMBO system with a (*μ* × *μ*) unit images. Since the span of *i* = 1, 2, … *μ* and *j* = 1, 2, …, *μ*, thus the averaged spectral and cross-spectral techniques can be applied similar to Eqns (13) and (14), but using spectral estimates instead of the true ones. Therefore, $F\left(z_{1}^{\frac{1}{D}},z_{2}^{\frac{1}{D}}\right)$ and similarly $H_{i,j}\left(z_{1}^{\frac{1}{D}},z_{2}^{\frac{1}{D}}\right)$ can be estimated iteratively using the equation,

(16) $$\sum_{i=1}^{\mu }\sum_{j=1}^{\mu }{\hat{C}}_{G_{i,j}H_{i,j}^{*}}(z_{1},z_{2})=F\left(z_{1}^{\frac{1}{D}},z_{2}^{\frac{1}{D}}\right)\sum_{i=1}^{\mu }\sum_{j=1}^{\mu }{\hat{C}}_{H_{i,j}H_{i,j}^{*}}\left(z_{1}^{\frac{1}{D}},z_{2}^{\frac{1}{D}}\right)+\sum_{i=1}^{\mu }\sum_{j=1}^{\mu }{\hat{C}}_{V_{i,j}H_{i,j}^{*}}\left(z_{1}^{\frac{1}{D}},z_{2}^{\frac{1}{D}}\right)$$

where Ĉ_XY__*_(*z*_1_, *z*_2_) = *X*(*z*_1_, *z*_2_)Y*(*z*_1_, *z*_2_) is an estimate of the cross-spectra between *X*(*z*_1_, *z*_2_) and *Y*(*z*_1_, *z*_2_). $\frac{1}{D^{2}}$ is here not considered since it is only a scaling factor.

For large values of (*μ* × *μ*) unit images, the last summation error term of Eqn. (16), is nothing but the mean value of an i.i.d signal which has a zero mean [13][14]. In this situation, we will have,

(17) $$\hat{F}\left(z_{1}^{\frac{1}{D}},z_{2}^{\frac{1}{D}}\right)\approx \frac{\sum_{i=1}^{\mu }\sum_{j=1}^{\mu }{\hat{C}}_{G_{i,j}H_{i,j}^{*}}\left(z_{1},z_{2}\right)}{\sum_{i=1}^{\mu }\sum_{j=1}^{\mu }{\hat{C}}_{H_{i,j}H_{i,j}^{*}}\left(z_{1}^{\frac{1}{D}},z_{2}^{\frac{1}{D}}\right)}$$

**Figure 6.**
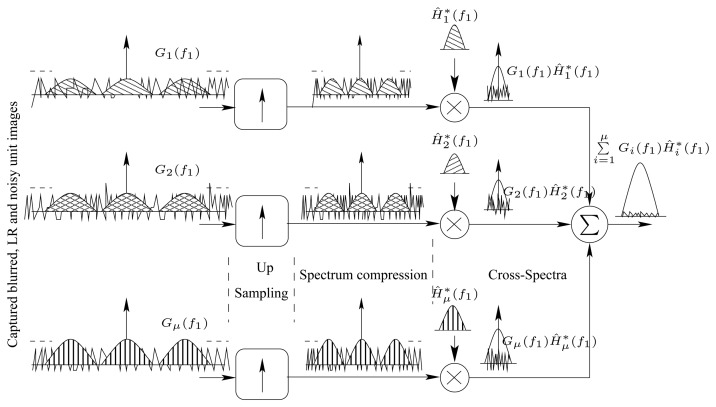
A spectral diagram showing how cross-spectra are estimated in the case of a 1-d signal

Fig. 6 is a spectral diagram illustrating how signals' cross-spectra are estimated so that the original image can be restored while minimizing the effect of additive noise and aliasing terms resulting from down-sampling. A similar diagram can be drawn for the PSF function.

## 4. Blind Image Restoration Algorithm

Blind image restoration is to reconstruct an original image from degraded, blurred and noisy observation(s) without any knowledge about either the original image or the degradation process, which includes the noise and the blur PSF [15]-[22].

**Figure 7.**
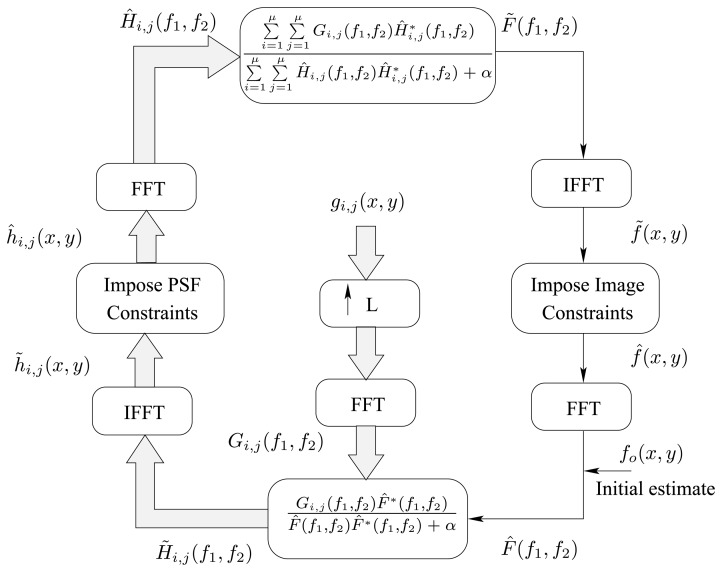
Blind Image Restoration Algorithm

Using the analysis carried out in the previous section, we have developed a blind image restoration algorithm capable of restoring the original image using only the captured, noisy and LR unit images. The proposed algorithm is given in Fig. 7.

During the restoration process, the algorithm will only impose two constraints on the restored image and the PSFs: the positivity and the support region. The two constraints can be described mathematically as follows,

- For the image
  (18) $$\hat{f}(x,y)=\{\begin{array}{cc} |\tilde{f}(x,y)|, & <x,y>\in \text{support region}[LM,LN]\\ 0 & \text{otherwise} \end{array}$$
- For the PSFs
  (19) $${\hat{h}}_{i,j}(x,y)=\{\begin{array}{cc} \left|{\tilde{h}}_{i,j}(x,y)\right| & <x,y>\in \text{support region}[\ell ,\ell ]\\ 0 & \text{otherwise} \end{array}$$

where, *L* ≥ *D* is the up-sampling factor needed to restore the high resolution image, (*LM* × *LN*) is the size of the restored image which can be greater or equal to the size of the original image (*M* × 
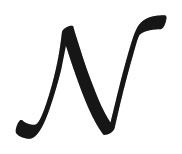
), and (ℓ × ℓ; is the size of the estimated PSF.

For the purpose of implementations and to evade the division by zero in Eqn. (17), a small value *α* can be added to the denominator (Fig. 7). Finally, the up-sampling is done by zero-padding in the spatial domain between the image samples, then Fast Fourier Transform (FFT) is applied and in the Fourier domain, a single spectrum is then taken out of the repetitive spectrums using a low pass filter with cutoff frequency $\left(\frac{\pi }{L}\right)$ and zeroing the rest of the spectrum. Finally, the inverse FFT (IFFT) is used to return to the image domain. Notice that the zero-padding must be such that the zero frequency components remain the same and that zero-padding is applied to both positive and negative frequencies. The whole process is symbolized by (↑ *L*) in Fig. 7. Other interpolation techniques could also be used.

## 5. Experimental Results and Discussion

**Figure 8.**
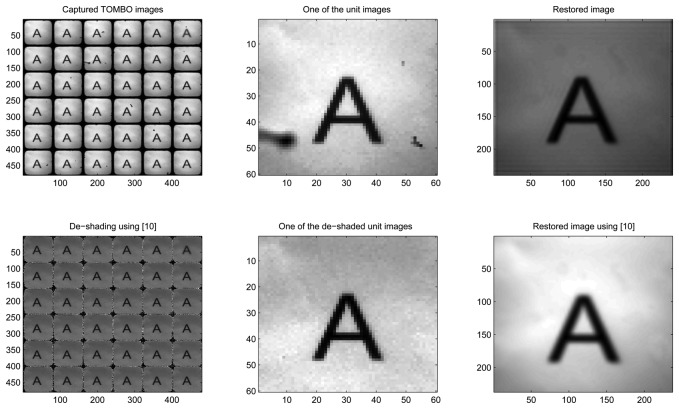
Experimental, no added noise, 6 × 6 lenses

In this section, we applied the proposed method to restore high resolution images from LR noisy images captured by an experimental TOMBO imagers. Captured images for a letter ‘A’ picture (Fig. 8), were provided by [3]. The letter ‘A’ picture was located 285 *mm* from the TOMBO imager, which comprises (10× 10) imaging units. Each unit has (60 × 60) pixels and each pixel is 6.25 *μm*× 6.25 *μm*. The microlens array has the following characteristics: 1.3 *mm* focal length, 0.5 *mm* diameter of aperture, and a 0.5 *mm* pitch for the microlens array.

To assess the robustness of the proposed method in the presence of noise, a zero mean correlated and uncorrelated Gaussian noise is added manually to the captured images. We consider the restoration of the original image for the following four different cases: (i) no added noise (ii) additive external noise (iii) additive internal and external noise (iv) additive external correlated noise. Information about test input data and output images can be found in Table 1.

The external noise is directly added to the captured images, while the internal noise is increased by altering the black, *B*(*x*, *y*), and white *W*(*x*, *y*) images (Eqns. (1) and (2)).

In Fig. 8 (no added noise), our blind restoration method is seen to perform slightly better than the pixel rearrangement method.

**Figure 9.**
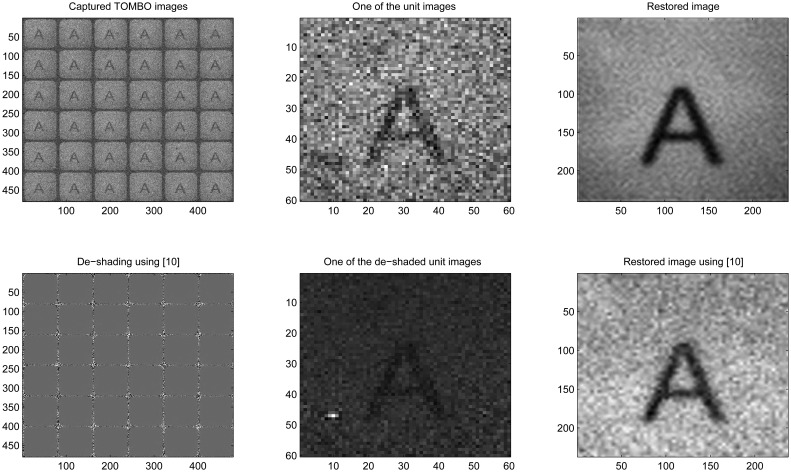
Experimental, External noise, 6 × 6 unit images

In Fig. 9, under external noisy conditions, one can see that our method performs better than the pixel rearrangement method.

**Figure 10.**
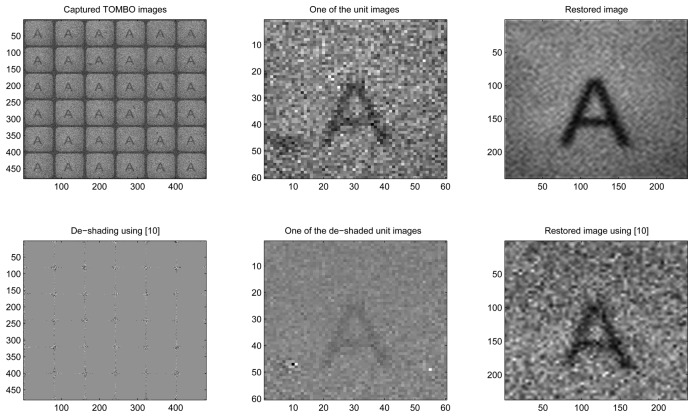
Experimental, Internal and external noise, 6 × 6 unit images

In Fig. 10, under internal and external noisy conditions, one can see that the de-shading pre-processing step further degrades the captured images. As a result, the pixel rearrangement method was unable to restore the original image, while our proposed method is able to. We have considered the effect of the internal noise because the conditions under which the black and white calibration images have been captured can change. In other words, when a new image is captured, the system will encounter noise levels and nonlinearities that are different from the ones encountered when calibrating the system using the black and white images.

**Figure 11.**
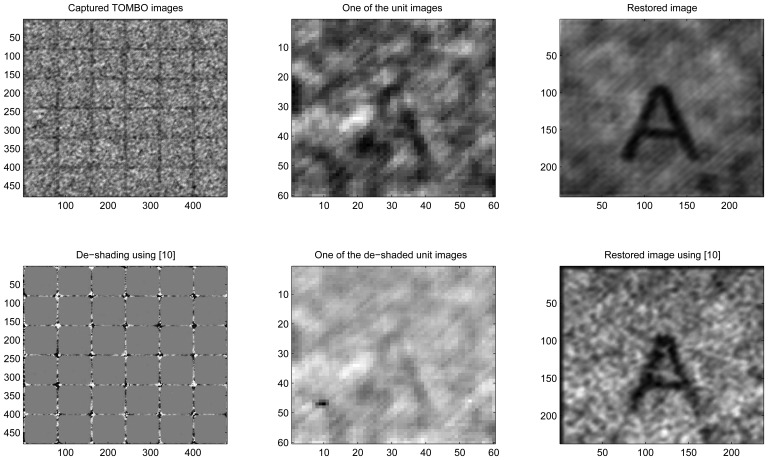
Experimental, External correlated noise, 6 × 6 unit images

In Fig. 11, under an external correlated noise, it is clear that the pixel rearrangement method is unable to restore the original image accurately, while our method can restore the original image. The reason behind the lower performance of the rearrangement method is that correlated noise creates false peaks (spares) that significantly affect the alignment performance of the rearrangement method (Fig. 2). In contrast, in our method, spectral information is not affected by misalignments (shifts *δx* and *δy* in Fig. 2).

In summary, the results of Figs. 8, 9, 10 and 11 show that our spectral-based blind image restoration method can reconstruct the original image in noisy conditions. This is achieved whether the source of noise is internal or external, uncorrelated or correlated, and at low signal-to-noise Energy ratio (SNER) as seen in Table 1.

Note also, the impact of the de-shading pre-processing step in Figs. 8, 9, 10 and 11, with the values of pixels increasing especially at the periphery of each unit image.

To compare the performance of our approach with the pixel rearrangement method, when the blur is significant and the down-sampling is high, a “Lena” image [23], *f*(*x*, *y*) of (*M* =256× 
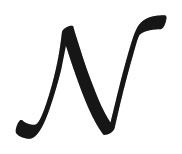
 =256; pixels is used to represent the original image. To accurately model real-world conditions, the original image is first convolved with (*μ* × *μ*) different PSFs representing the blur. A rotationally symmetric truncated Gaussian PSF (blur) combined with a motion-like PSF, ${\text{h}}_{o}^{\ell \times \ell }$, with standard deviation *σ* is used as a reference. From the reference PSF, all PSFs are generated such that, *h_i,j_*(*x*, *y*) = *h_o_*(*x*, *y*) + *r_i,j_*(*x*, *y*) where *r_i,j_*(*x*, *y*) is a random variable uniformly distributed between [−*δ,δ*]. To model a captured TOMBO unit image *g_i,j_*(*x*, *y*), the resultant blurred image plus the noise is then down-sampled (↓, *D*) in a way that mimics the function of the imager. Mathematically we have,

(20) $$\begin{array}{lll} g_{i,j}(x,y) & = & \frac{1}{D^{2}}\sum_{\overset{\prime }{x}=Dx}^{(x+1)D-1}\sum_{\overset{\prime }{y}=Dy}^{(y+1)D-1}z_{i,j}(\overset{\prime }{x},\overset{\prime }{y}),\\ z_{i,j}(\overset{\prime }{x},\overset{\prime }{y}) & = & h_{i,j}(\overset{\prime }{x},\overset{\prime }{y})**f_{i,j}(\overset{\prime }{x},\overset{\prime }{y})+\upsilon_{i,j}(\overset{\prime }{x},\overset{\prime }{y}) \end{array}$$

Simulation parameters are given in Table 2.

**Figure 12.**
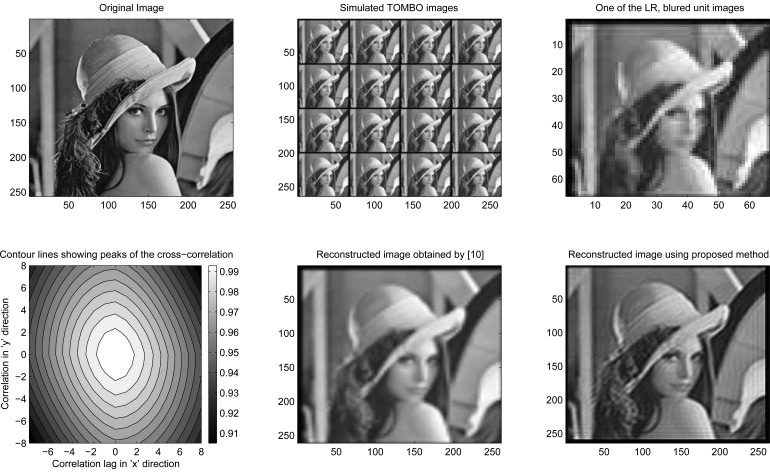
Simulation, no added noise, 4 × 4 unit images

From Fig. 12, it is clear that with realistic PSFs, our restoration method outperforms the pixel rearrangement method. This is because the pixel rearrangement method is unable to align the captured unit images for the simulation parameters in Table 2. To illustrate this, the cross-correlation function between a reference image and a unit image is represented by the contour lines in Fig 12. Note that there is a single peak at (*x* = 0, *y* = 0). However, there should be at least 7 peaks due to the motion-like PSFs since each has (7 × 7) coefficients. Instead, only a single peak is recognized by the pixel rearrangement method, leading to the observed blurred output image, which requires inverse filtering.

## 6. Conclusions

A spectral-based blind image restoration method for thin TOMBO imagers is described and analyzed. The proposed method does neither require conventional de-shading or pixel rearrangement as these operations are performed automatically through the blind restoration process. As a result, errors associated with misalignment, nonlinearities, singularities and high pixel values are overcome. Experimental results demonstrate that the proposed method can reconstruct the original image wether the source of additive noise is internal or external, uncorrelated or correlated. In contrast to conventional pixel rearrangement method, it is able to restore the original at SNER lower than 3dB. The proposed method can also be extended to color images.

## Figures and Tables

**Table 1.** Test input data

Figure	*μ* × *μ* used	*M* × *N*	SNR	ℓ	↑ *L*	*LM* × *LN*	*α*	# of Iterations
Fig. 8, No added noise	6 × 6	60 × 60	-	3	4	240 × 240	0.01	30
Fig. 9, External noise	6 × 6	60 × 60	7 dB	3	4	240 × 240	0.01	30
Fig. 10, Internal and external noise	6 × 6	60 × 60	40 dB (In) and 7 dB (Ex)	3	4	240 × 240	0.01	30
Fig. 11, External correlated noise	6 × 6	60 × 60	2.8 dB	3	4	240 × 240	0.01	30

**Table 2.** Simulation input data

Figure	ℓ, *σ*	↓*D*	SNER	*μ* × *μ* unit images	*M* × *N*	↑*L*	*LM*×*LN*	*α*	# of Iterations
Fig. 12	7, 4	4	-	4 × 4	64 × 64	4	256×256	0.01	30
